# BCST-GCN: a skeleton-based spatiotemporal graph convolutional network with bidirectional cross-attention for pig behavior recognition

**DOI:** 10.3389/fvets.2026.1782396

**Published:** 2026-04-13

**Authors:** Haojie Chai, Weibo Zhan, Jianshuai Su

**Affiliations:** 1 College of Artificial Intelligence, Henan Institute of Science and Technology, Xinxiang, China; 2 College of Computer Science and Technology, Henan Institute of Science and Technology, Xinxiang, China

**Keywords:** graph convolutional network, pose estimation, pig behavior recognition, attention mechanism, DeepLabCut, BCST-GCN

## Abstract

To address the issues of weak inter-frame motion correlation and poor recognition robustness in video-based pig behavior recognition, as existing methods fail to fully exploit the spatiotemporal dynamic features of skeletons and can hardly capture fine behavioral details, this study proposes a skeleton-based spatiotemporal dynamic modeling method for pig behavior recognition. We use DeepLabCut (DLC) to accurately extract pig skeleton keypoints and construct the topological structure, streamline the ST-GCN by removing redundant network layers, and design an improved BCST-GCN model with a global-local self-attention BC module to dynamically reconstruct topological correlations, so as to effectively capture non-physical connections and complex spatiotemporal behavior characteristics. Experimental results show that the proposed framework can effectively recognize typical behaviors such as feeding, walking, lying, and dog-sitting posture, and the improved model yields 6.94%, 5.61%, and 6.88% increments in accuracy, precision, and recall respectively compared with the baseline model. The proposed method achieves accurate and efficient pig behavior recognition, solves the problems of weak temporal correlation and insufficient feature extraction in traditional models, and provides a reliable technical solution for intelligent monitoring in pig farming scenarios, supporting the intelligent upgrading of the breeding industry.

## 1 Introduction

With the rapid development of machine learning, especially deep learning technology, such methods have been widely applied in the field of smart livestock farming (1) and have been extensively adopted for pig behavior recognition (2). With their powerful data processing and pattern recognition capabilities, these techniques have greatly advanced the pig farming industry.

However, key processes in large-scale pig farming, including disease prevention, health monitoring, and behavior analysis, still heavily rely on manual labor. The high labor cost has restricted the economic efficiency of the industry, creating an urgent demand for automated, refined, and intelligent farming modes. The core to achieving this goal lies in the accurate and efficient recognition of pig behaviors. Within the framework of precision livestock farming, behavior monitoring (3) is of great significance for making timely and effective management decisions and improving animal welfare (4). Studies have shown that changes in animal behavior (5, 6) are closely related to their health and welfare status, and pig behavior can effectively reflect their physical condition (7, 8). Typical behaviors such as lying, standing, and walking can directly indicate health status: for instance, pigs often maintain a sternal recumbent posture at low temperatures (9), a lateral recumbent posture at high temperatures (10), and a sitting posture when suffering from respiratory distress (11).

In the field of pig behavior recognition, traditional machine learning methods were first applied. Huang et al. (12) proposed a machine vision-based method for group-housed pig recognition. The method extracted Gabor and Local Binary Pattern (LBP) features from top-view images, generated feature vectors through Principal Component Analysis (PCA) dimensionality reduction, and conducted classification experiments using Support Vector Machines (SVM), exploring the application of traditional machine learning in pig behavior recognition. On this basis, deep learning has further advanced the field with its excellent end-to-end feature learning capability. Xiao et al. (13) combined YOLOX (You Only Look Once X) with the CowbodyNet feature extraction network for individual cow recognition, achieving 94.43% recognition accuracy on their dataset, which confirms the considerable feasibility and potential of deep learning methods in animal behavior recognition.

The continuous technological evolution from traditional machine learning to deep learning has continuously driven the transformation of animal behavior monitoring methods. Empowered by the strong capabilities of deep learning, computer vision technology has developed rapidly (14), introducing a new technological paradigm for animal behavior monitoring and gradually replacing traditional methods such as manual observation and wearable sensors. As a core supporting technology for computer vision tasks, deep learning can automatically extract effective feature representations from massive data through end-to-end learning, greatly reducing reliance on manual feature engineering and intervention (15–18). Relying on this advantage, research on deep learning-based animal behavior recognition has made remarkable progress (19, 20). For example, Ali Alameer et al. (21) adopted an improved YOLO (You Only Look Once) deep learning model integrated with a residual network structure to effectively identify and quantify head-tail contact behaviors among pigs; Heseker et al. (22) used deep learning algorithms to automatically monitor pig screaming, tail-biting events, and identify tail-biting individuals.

Currently, computer vision-based action recognition methods are mainly divided into two categories: image-based methods and skeleton-based methods. Image-based methods take RGB (red-green-blue) data as direct input (23, 24), but their performance is easily affected by environmental factors such as color variation, cluttered backgrounds, and lighting conditions. Norouzzadeh et al. (25) tested various deep neural networks on image datasets and found that single images cannot reliably distinguish visually similar behaviors such as walking and running. Animal behavior is continuously and dynamically evolving, rather than static. To overcome the limitation that traditional static image-based methods do not fully utilize temporal features of behaviors, Yang et al. (26) proposed a method that dynamically calculates spatial information from the udder region. Considering temporal characteristics, the method computes motion intensity and occupancy index through optical flow analysis within the udder region, enabling the differentiation of visually similar behaviors such as suckling. Gao et al. (27) employed the VGG16 model combined with a temporal feature extractor to extract spatial appearance features and motion features from each input frame, and introduced a spatial–temporal attention module to identify agonistic behaviors in group-housed pigs. Keceli et al. (28) used a Bidirectional Long Short-Term Memory (BiLSTM) network to predict calving dates and utilized a boosted tree classifier to forecast calving behavior 8 h in advance, thereby realizing calving behavior recognition. Although image-based deep learning methods can characterize the continuous changes of behaviors by introducing temporal modules into Convolutional Neural Networks (CNNs), such methods still rely only on image appearance features and fail to fully exploit the spatial structural information and temporal dynamic features of skeletons, making it difficult to achieve accurate modeling of behavioral essence. To address this shortcoming, many researchers have begun to utilize the internal dynamic features of skeletons to provide a finer-grained basis for behavior analysis and conduct animal behavior recognition tasks. As shown in the Spatial–Temporal Graph Convolutional Network (ST-GCN) proposed by Yan et al. (29), skeleton data can be constructed into a topological graph to represent human skeleton sequences, which are then converted into feature vectors for human action modeling. Since both human and animal behaviors depend on the movement of skeletal joints, the skeleton modeling approach designed for humans is also applicable to animal behavior analysis such as livestock and poultry.

Inspired by this, Li et al. (30) transferred the Part Affinity Fields method originally used for human pose estimation to dairy cows to extract skeleton information, enhancing the model’s attention to the cow itself, reducing dependence on background and appearance features, and thereby improving lameness detection accuracy. Tseng et al. (31) developed the ADD-LSTM system, which fuses image appearance features and skeletal structure information. By integrating image processing, skeleton detection, and long short-term memory (LSTM) networks, the method achieved high-precision analysis of dolphin behaviors. Although skeletal information was adopted, the model essentially belongs to non-graph convolutional networks, resulting in insufficient capability in capturing structural features of skeletons. Hua et al. (32) proposed a lightweight architecture that combines keypoint extraction based on YOLOX-Pose with the PoseC3D network capable of spatiotemporal feature modeling, realizing efficient and accurate recognition of typical behaviors in dairy cows. However, the differential demands of various behavioral recognition tasks on different joints were not considered—for instance, lameness detection requires enhanced attention to limb joints, while resting state recognition relies more on refined attention to the trunk. Yin et al. (33) employed the SPPCSPC module. Although it captures the global posture and local joint details of pigs through multi-scale feature fusion, it suffers from high computational overhead.

Although previous methods have improved the recognition accuracy of dynamic behaviors of animals such as pigs, cattle and sheep by using graph convolutional structures and introducing temporal correlation information, in pig movement behavior analysis, existing methods still fail to fully focus on the relationships between adjacent local joints. Meanwhile, they ignore the potential coordinated movement relationships between non-directly connected joints. In addition, the models have high computational overhead, which makes it difficult to meet the efficient deployment requirements in practical farming scenarios. To address these problems, this paper introduces a dynamic adaptive module in the process of topological modeling. It can not only accurately capture the dynamic changes in the relative positions and temporal correlations of joints, but also effectively mine the coordinated movement patterns between long-distance joints. Thus, the spatiotemporal dynamic characteristics of behaviors can be more comprehensively described, and the behavior recognition accuracy is further improved.

Given the above limitations of image-based methods, this study does not adopt schemes that only consider image appearance features, but instead selects skeleton-based approaches, aiming to achieve higher robustness and accuracy.

This study focuses on one core direction in the field of behavior recognition: skeleton-based action recognition. In skeleton-based action recognition, pose estimation is a necessary prerequisite for obtaining target keypoints. However, there is currently a lack of publicly available pig pose data and corresponding image data. Therefore, pose estimation will become an indispensable part of this study. We will systematically elaborate from two dimensions: pose estimation and action recognition.

Accordingly, this paper proposes a skeleton keypoint-based method for pig pose estimation and behavior recognition, named BCST-GCN. The method consists of two core tasks: pose estimation and behavior recognition. The main research content revolves around three key modules: the construction of core keypoints, the global and local feature learning module (BC), and the layer-optimized Spatial–Temporal Graph Convolutional Network (ST-GCN).

The main contributions of this study are summarized as follows.

Although previous studies have considered the spatial–temporal variation characteristics of dynamic behaviors, overly rigid assumptions about skeleton topology and insufficient attention to non-physical connections remain limitations. To this end, we present the following contributions:

1. This paper proposes a novel intelligent analysis tool, BCST-GCN, which combines a pose estimation network with an improved spatial–temporal network for pig behavior recognition.
2. This paper adopts pose estimation technology to identify pig skeleton keypoints, providing basic data for subsequent in-depth analysis. Furthermore, inspired by human skeleton-based action recognition, we use ST-GCN as the baseline, which is more powerful in describing non-Euclidean data such as skeleton graphs. A graph convolution method fusing global–local self-attention is proposed.
3. In this work, a dual-path interaction mechanism is proposed to enhance skeleton modeling. The global self-attention dynamically constructs potential dependency topologies among joints, breaking the static constraints of traditional adjacency matrices and strengthening cross-node interaction modeling. The local self-attention quantifies the coupling coefficients of first-order neighborhood joints based on physical connection strength to achieve fine-grained local dynamic perception. On this basis, an improved BCST-GCN network is constructed by reducing the number of ST-GCN layers, further improving the accuracy and efficiency of behavior recognition.

**Figure 1:**
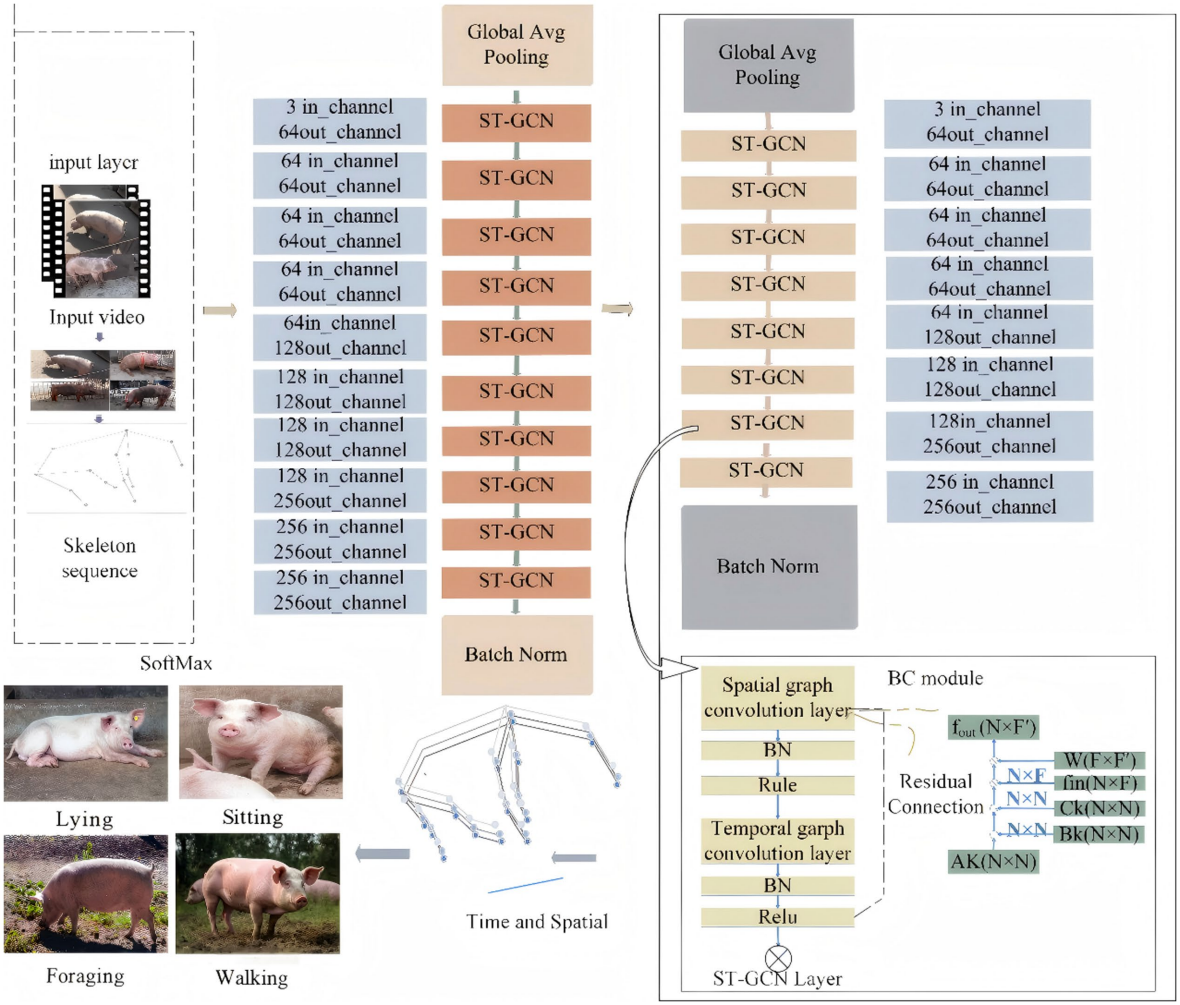
General structure of the model.

The architecture of the proposed method is clearly illustrated in Figure 1. Specifically, a pose estimation algorithm is used to accurately obtain the keypoints of the pig’s body and generate a skeleton graph accordingly. This skeleton graph, as critical data, is input into the Graph Convolutional Neural Network for subsequent processing. In the graph convolutional network, the BC module is innovatively integrated, which further captures the dynamic topological structure and enhances non-physical connections. Finally, the action recognition task is completed via the SoftMax operation. The method focuses on the dynamic changes of the skeleton, breaks away from the constraints of a fixed skeleton model, and can thus capture action features more accurately.

## 2 Related work

### 2.1 Posture estimation

Deep learning has revolutionized computer vision since 2014. Among its advancements, groundbreaking progress enhances practicality and accuracy, with 2D human-pose estimation benefiting significantly from methodological improvements. Leveraging datasets like Common Objects in Context (COCO) (34) and Human Pose Dataset (MPII) (35), human pose estimation has rapidly evolved across diverse applications. Given the kinematic similarities in limb movements between animals and humans, alongside the intrinsic value of animal behavior research, techniques and insights from human pose estimation have been adapted to advance animal behavior recognition (36).

The MPII Human Pose dataset contains data in units of ten thousand. DeepLabCut (37) utilizes transfer learning and pre-trained networks, achieving human-level annotation accuracy with approximately 200 training frames per image. Experimental results demonstrate its robust tracking performance, with low testing errors in tasks such as mouse odor navigation and fruit fly behavior analysis. Wiltshire et al. (38) applied DeepLabCut to video data of wild chimpanzees and bonobos for pose estimation and behavior tracking, establishing novel methodologies for wildlife behavior research. Fang et al. (39) and Nasiri et al. (40) utilized DeepLabCut as a keypoint detector for pose estimation in broiler chickens. Demonstrating superior performance, DeepLabCut enables multi-animal pose estimation and effectively extracts kinematic data in complex visual environments, making it widely adopted in animal posture analysis. Therefore, this study employs DeepLabCut to focus on 2D animal pose estimation research.

### 2.2 Animal behavior recognition

Human action recognition tasks typically involve identifying the behavioral categories present in a video. Due to the rich information contained in videos, different methods model and analyse behaviors using diverse perspectives, such as appearance, optical flow, and skeletal data. Skeletal-based action recognition methods can be broadly categorized into two groups: traditional manual feature-based approaches (41–43) and deep learning-based methods (44–49).

In recent years, some researchers have begun to utilize deep learning methods to extract skeletal sequence information from video targets for behavior recognition. These recognition methods are categorized into three main groups: Recurrent Neural Networks (RNNs)- approaches, Convolutional Neural Networks (CNNs)- based approaches, and graph convolutional networks based approaches. Convolutional Neural Networks exhibit limited capabilities in extracting critical features from skeletal diagrams. While LSTM-based skeletal recognition methods account for the temporal and spatial properties of networks, their architectures are structurally complex. Graph convolutional networks, which model skeletal data using graph structures, precisely capture the spatial relationships between human joints, achieving superior performance in action recognition compared to prior methods. Advances in human behavior research have developed possibilities for advancing animal behavior recognition. Fuentes et al. (50) proposed a hierarchical bovine behavior recognition method based on deep learning, integrating spatiotemporal information and frame-level appearance features. Results demonstrated that the system effectively identifies 15 hierarchical bovine behaviors in day-night environments. Li et al. (30) utilized ST-GCN in livestock behavior recognition to extract features from bovine skeletal joint coordinates and trajectories, capturing skeletal connectivity patterns and temporal dynamics for multi-target pose estimation. Liu et al. (51) combined convolutional neural networks with recurrent neural networks to extract spatiotemporal features for identifying and localizing tail-biting behaviors. The overall method achieved an 89.23% accuracy in identifying and localizing tail-biting behaviors.

However, although existing methods have adopted various network models and introduced temporal correlation information to improve the recognition accuracy of dynamic behaviors in animals such as pigs, cattle and sheep, they still fail to fully capture the dynamic changes in the relative positions and correlations of joints over time when analyzing pig movement. In addition, they ignore the potential cooperative relationships between non-directly connected joints. To address these limitations, this paper introduces a dynamic adaptive module into the topological modeling process. This module can accurately capture the dynamic changes in the relative positions and temporal correlations of joints, and effectively mine the cooperative motion patterns between distant joints. In this way, it can comprehensively characterize the spatiotemporal dynamic characteristics of behaviors and further improve the recognition accuracy.

## 3 Method

### 3.1 Data acquisition and processing

The hardware setup comprised an NVIDIA GeForce RTX 3060 GPU, AMD Ryzen 7 5800X CPU, and 32GB RAM. Software dependencies included Python 3.8 (PyTorch framework) and DeepLabCut v2.3.7, supplemented by NumPy 1.19.5, OpenCV 4.5.3, and Matplotlib 3.4.3.

The pig behavior video dataset constructed in this study was collected from publicly available online resources and used only for non-commercial academic research. All data were obtained through manual retrieval, screening, and organization without web crawlers or automated bulk download tools, in strict compliance with the usage terms and copyright regulations of relevant platforms. The copyright of the videos belongs to the original creators. This study only uses the data for model training and scientific analysis without involving redistribution or commercial application of the original videos. To ensure balanced and diverse behavior categories, manual retrieval was conducted using keywords including walking, lying, standing, feeding, and canine-like sitting. After collection via platforms such as Baidu, Bing, and Google, the preliminary video materials were obtained through deduplication, invalid content filtering, and manual verification.

This study takes finishing pigs of the Large White breed as the research subjects, and the dataset covers both indoor and outdoor breeding environments. The data were collected from online public platforms, and no field shooting experiments were performed, so the shooting angles and distances of the original videos cannot be accurately verified. To ensure the accuracy of behavior recognition and the quality of keypoint annotation, strict screening was conducted on the original videos according to unified criteria during data preprocessing, and video clips captured under natural daylight were preferentially retained. Meanwhile, to facilitate clear annotation of skeletal keypoints and improve the localization accuracy of joints, videos with lateral views and a small number of top-down views were mainly retained, while clips that were too close to fully display keypoints, as well as samples with blurry frames, severe occlusions, and viewing angles unfavorable for keypoint annotation, were removed. The dataset initially contained 421 raw video clips. After two-stage strict screening to remove clips that were too short, of low quality, or with insignificant posture and motion features, 197 high-quality videos were retained, all with a resolution of 1920 × 1,080 and an average duration of approximately 9.2 s.

**Figure 2:**
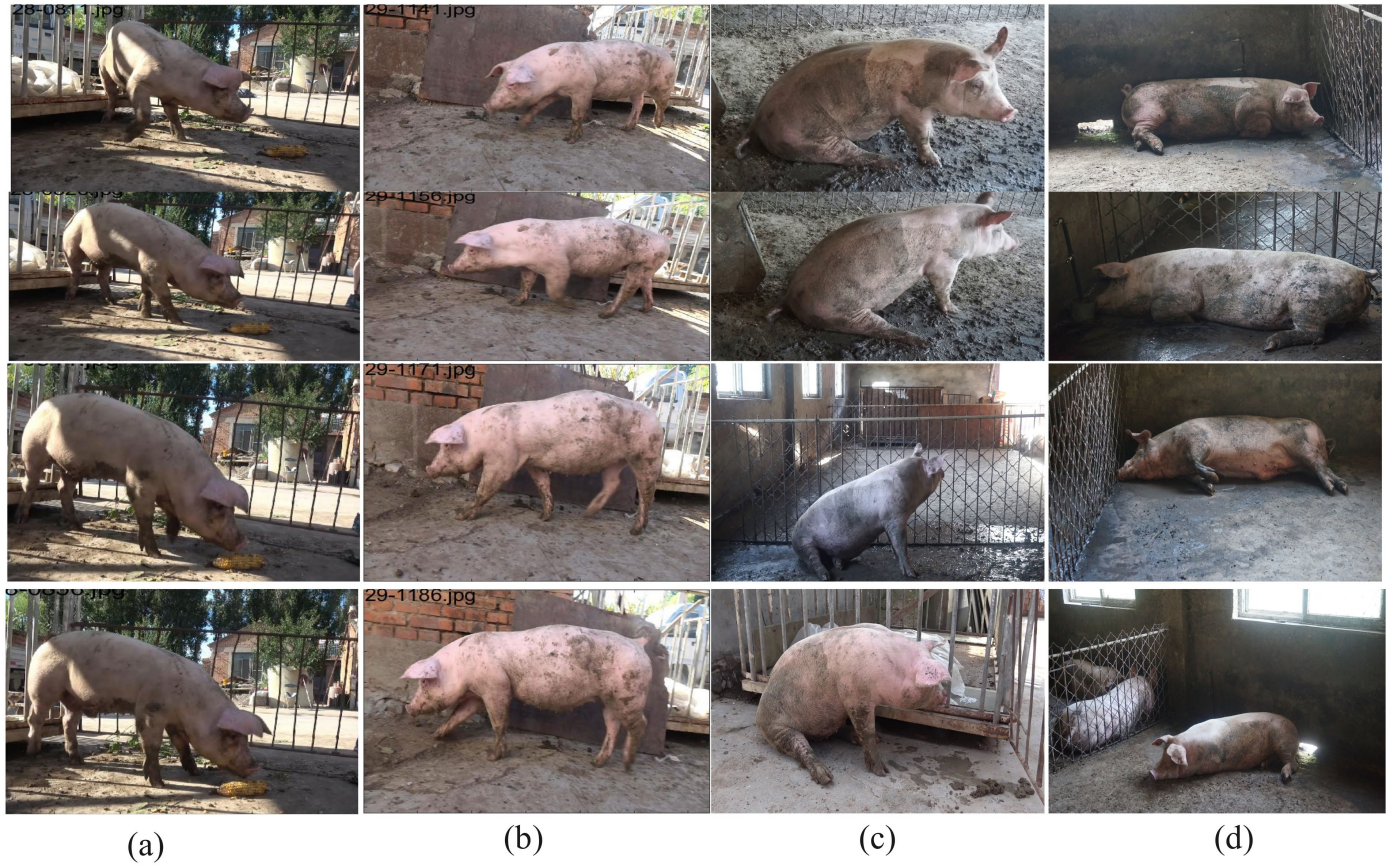
Partial dataset presentation. **(a)** Feeding consecutive frames, **(b)** walking consecutive frames, **(c)** canine sitting consecutive frames, **(d)** lying consecutive frames.

To construct training samples suitable for temporal behavior recognition, video clips were extracted at a fixed frame rate of 25 fps. After manual screening, approximately 180 valid clips covering behaviors such as feeding, walking, sitting, and lying were obtained, with an average duration of about 6 s. While preserving temporal information, uniform sampling was applied to generate about 6,230 representative key frames, which constitute the core training set (part of its visualization is shown in Figure 2). To enhance model robustness and environmental adaptability, data augmentation strategies including random occlusion, joint erasure, background disturbance, and Gaussian noise were employed during training. Throughout the augmentation process, the topological structure of key points was maintained to improve the model’s generalization capability in complex breeding scenarios.

**Figure 3:**
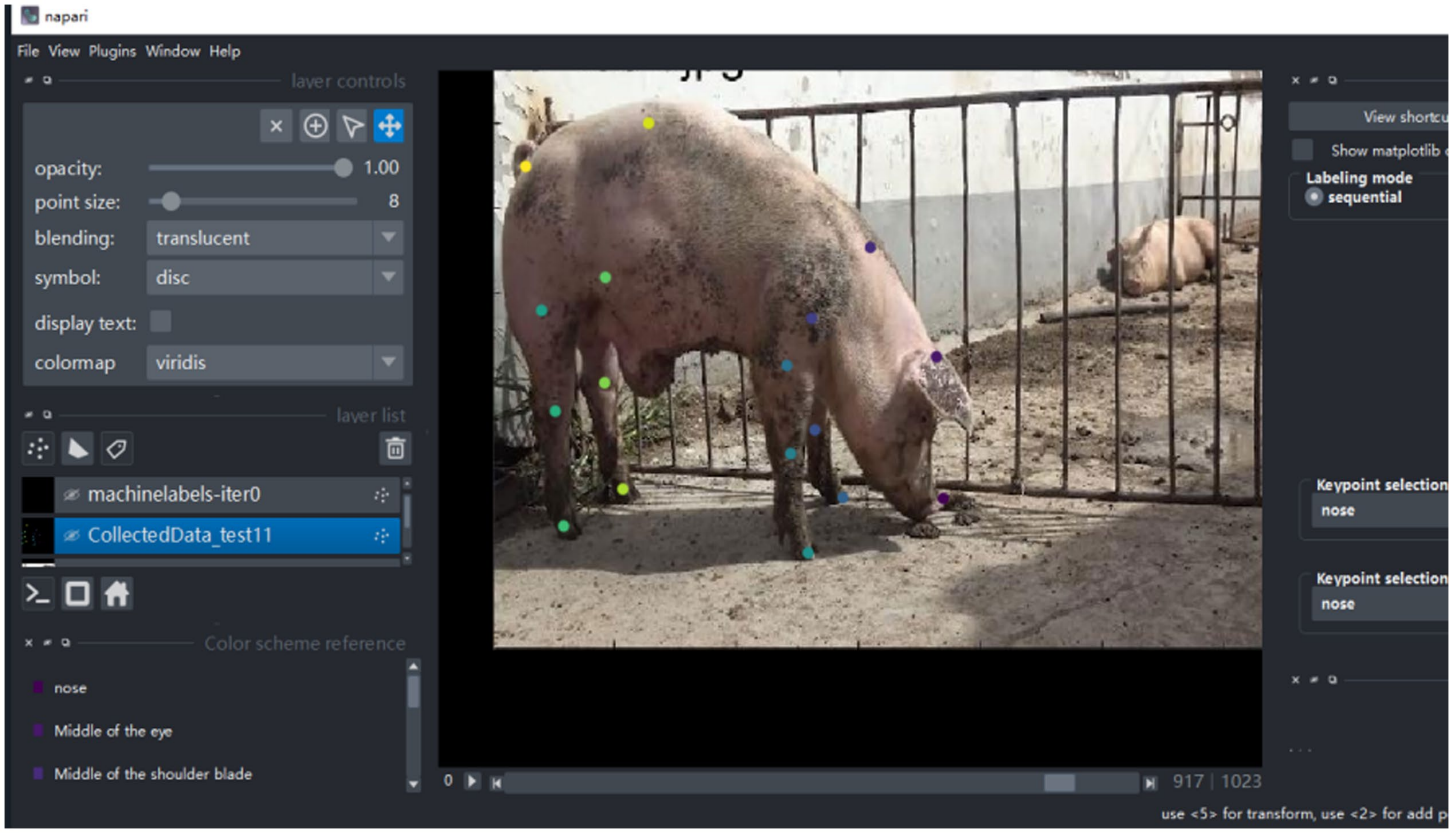
Example of key point annotation using the Napir tool.

This study finally retained 180 video samples. Videos with prolonged stillness or lacking significant motion variations were excluded to ensure sufficient pose and behavioral diversity, meeting the requirements for deep learning model training. All videos had a resolution of 1920 × 1,080. The data preprocessing pipeline comprised four sequential stages: video data capture, video frame segmentation, image data filtration, and video frame data annotation. During the annotation stage, we labeled key anatomical keypoints of pigs, including the head, trunk, limbs, and tail. This study used the Napir tool for keypoint annotation, as shown in Figure 3, which significantly improved annotation quality and efficiency. Finally, approximately 6,230 valid images were extracted from the 180 screened videos. To enhance model robustness and accuracy, data augmentation was applied to these valid images, resulting in 18,690 augmented images. The entire dataset was then divided into training, validation, and test sets with a ratio of 7:2:1. Detailed information of the dataset is provided in Tables 1, 2.

**Table 1:** Dataset division situation.

Dataset	Number of images
Train set	13,083
Validation set	3,738
Test set	1,869

**Table 2:** Classification of data sets by category.

Dataset	Foraging	Walking	Lying	Sitting
Training set	4,210	3,490	2,690	2,693
Validation set	1,200	1,000	770	768
Test set	600	500	380	389

Pig skeleton extraction serves as a critical method for recognizing the postures of moving pigs. This study adopts a lateral perspective in skeletal design, referencing the largest existing wild animal pose dataset, Animal Pose 10 k. Research indicates that lateral views demonstrate superior performance in conveying skeletal details. Skeletal information comprises keypoints and their connecting lines, simulating the structure of actual skeletons to efficiently depict pig postures and provide reliable support for behavior recognition. Pig skeleton extraction serves as a critical method for recognizing the postures of moving pigs. This study adopts a lateral perspective in skeletal design, referencing the largest existing wild animal pose dataset, Ap-10 k. Research indicates that lateral views demonstrate superior performance in conveying skeletal details. Skeletal information comprises keypoints and their connecting lines, simulating the structure of actual skeletons to efficiently depict pig postures and provide reliable support for behavior recognition.

**Figure 4:**
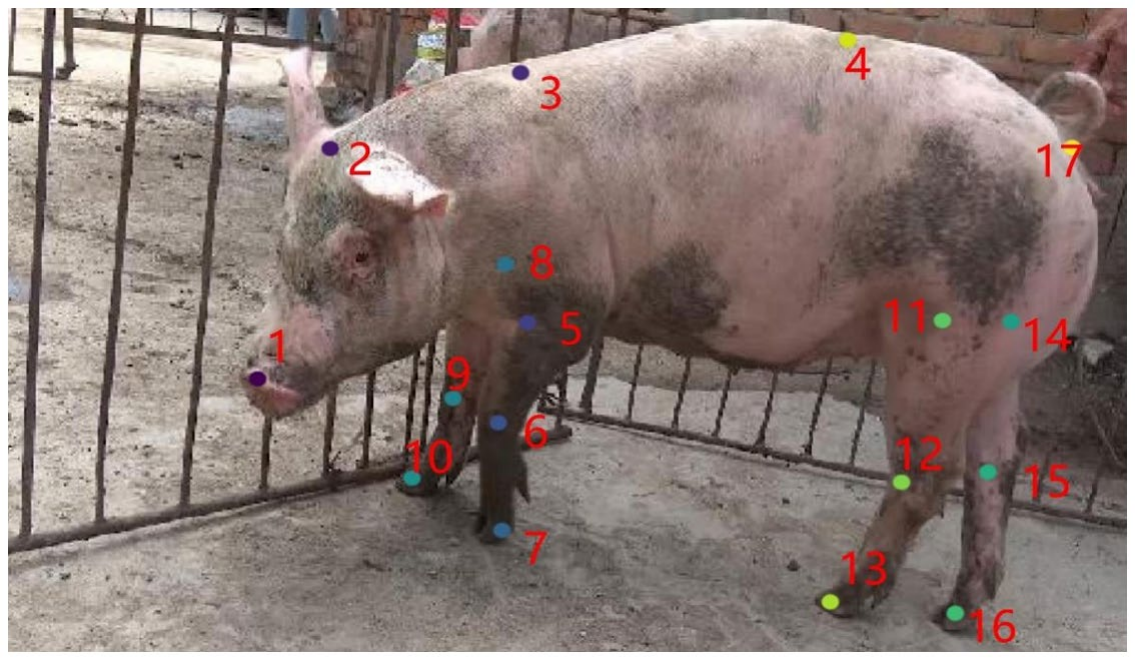
Keypoints presentation.

The design of pig skeletal keypoints and connecting relationships forms the foundation for extracting skeletal features. In this study, 17 keypoints and 16 pairs of connecting relationships were designed based on pig skeletal characteristics and the actual positions of body organs, as shown in Figure 4. These keypoints are numbered as follows: 1 (Nose), 2 (Between the ears), 3 (Neck base), 4 (Shoulder midpoint), 5 (Left forelimb base), 6 (Left front knee), 7 (Left front hoof), 8 (Right forelimb base), 9 (Right front knee), 10 (Right front hoof), 11 (Right hindlimb base), 12 (Right hind knee), 13 (Right hind hoof), 14 (Left hindlimb base), 15 (Left hind knee), 16 (Left hind hoof), and 17 (Tail root). Critical regions, including the head, limbs, back, chest, and tail, were color-coded with distinct colors to visually differentiate anatomical features. These skeletal features provide critical feature support for accurately describing pig postures and behavior recognition.

### 3.2 Methods for estimating pig posture

DeepLabCut is a deep convolutional neural network architecture (52). It integrates two core algorithmic components for object recognition and semantic segmentation: a preprocessed Residual Network (ResNet) feature extraction module and deconvolutional layer architectures (53). The network utilizes weight parameters pretrained on large-scale object recognition benchmark datasets such as ImageNet, which have demonstrated exceptional performance in this benchmark test (54). Unlike conventional designs that place classification layers at the end of ResNet architectures, deconvolutional layers generate spatial probability density distributions by introducing visual information. To enhance network adaptability to specific tasks, the network weights are fine-tuned using training datasets composed of video frame sequences and corresponding landmark coordinate annotations. For pose estimation tasks, ResNet-50 replaces the original classification and average pooling layers with deconvolutional layers to upsample feature maps, generating spatial probability density distributions for keypoints. The model determines keypoint locations by selecting the pixel coordinates with the highest confidence, enabling direct mapping from input images to keypoint coordinates. During training, the model leverages pretrained ResNet-50 to extract features and optimizes the spatial distribution of keypoint predictions, improving robustness against single-target posture variations and background interference while addressing single-target behavior analysis requirements.

**Figure 5:**
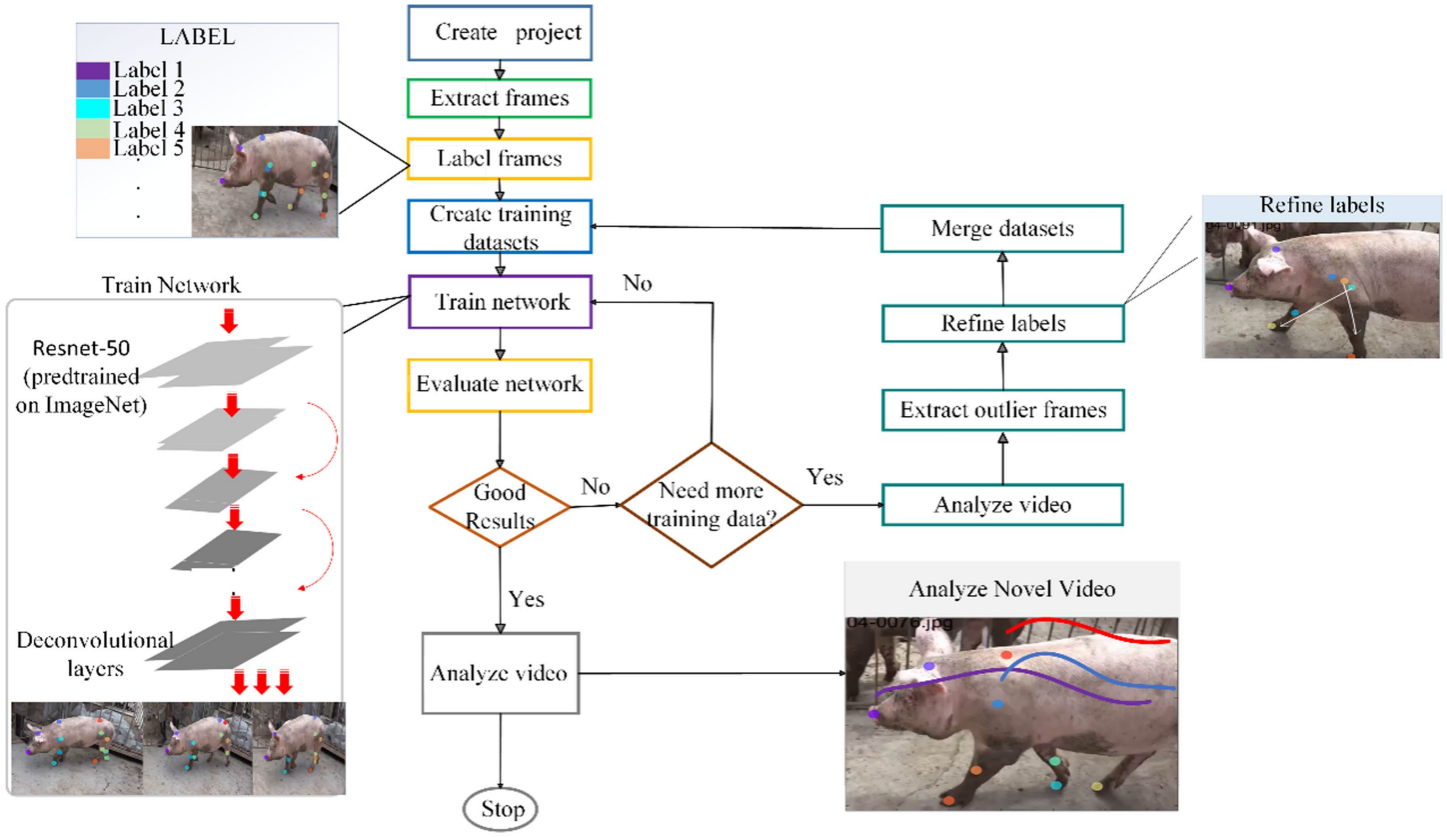
Flowchart of attitude estimation method.

This study employs a Residual Network as the backbone network for DeepLabCut. As shown in Figure 5, the research focuses on keypoint identification and tracking for single-target pigs, achieving precise localization of 17 keypoints and posture estimation. ResNet-50 utilizes residual blocks and skip connections to effectively address the gradient vanishing problem in deep networks, significantly enhancing feature extraction capabilities. This enables the network to accurately capture posture features of single-target pigs in complex environments, providing robust support for subsequent precise keypoint localization and posture estimation.

### 3.3 Pig behavior recognition model ST-GCN

**Figure 6:**
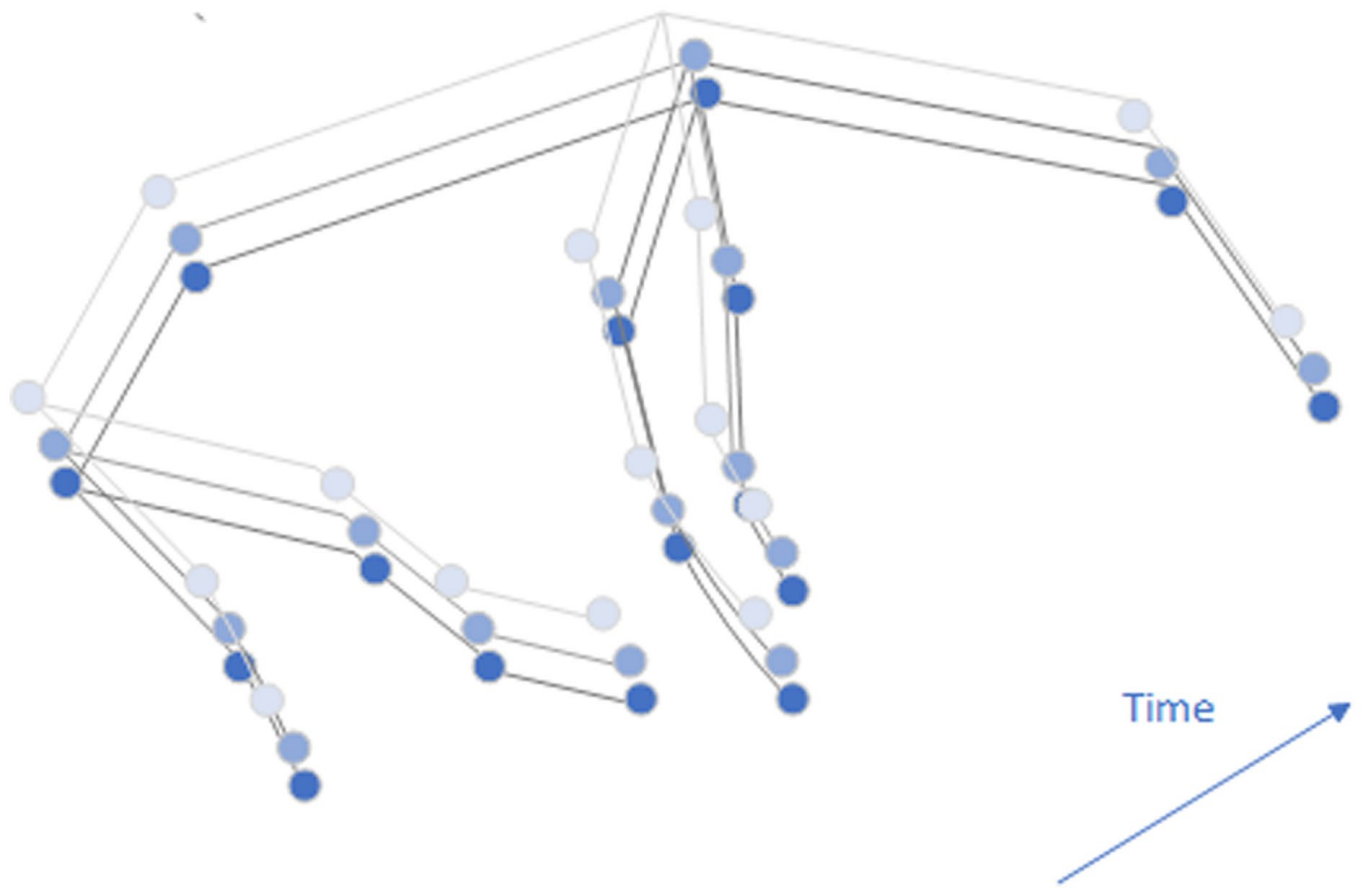
Diagram of a pig skeleton.

In spatiotemporal graph convolution units, the importance of internode edges is learned by using learnable edge weight parameters. The continuous joint information serves as the input feature for the ST-GCN network. This input feature undergoes Batch Normalization (BN) before being convolved through a series of ST-GCN unit layers, and finally the Global Average Pooling (GAP). The input features consist of a 4-dimensional matrix (N, C, T, V), where N represents the number of videos, C represents the joint features, T represents the number of keyframes, and V represents the number of joints. Construct space–time skeleton figure G = (V, E). Where, the point set V represents the joint set, and the edge set E includes space edge Es and time edge Et. Space side $E_{s}=\{v_{\mathrm{ti}}v_{\mathrm{tj}}|(i,j)\in H\}$ connects different key points and time sides $E_{t}=U_{i=1}^{n}\{(v_{\mathrm{ti}}v_{(t+1)i})|t=1,2\ldots ,T-k;k\in \{1,2,\ldots k\}\}$ in the same frame, and time side connects the same key points in different frames. The temporal and spatial structure of pig bone structure is shown in Figure 6.

Figure 6 shows a schematic of the skeleton. Blue dots represent pig skeletal joints, black lines represent spatial edges of pig joints, and light black lines represent temporal edges of the same joints in adjacent frames.

In order to extract high level semantic features, multiple ST-GCNs are stacked and combined with Fully Connected (FC), and the classification is completed by a fully connected layer. The spatial dimensions of the convolution operation formula in the above figure are as shown in Equation (1):


$$f_{\text{out}}=\sum_{k}^{K_{v}}W_{k}(f_{m}({\bar{A}}_{k}⊙M_{k}))$$ (1)

In Equation (1), *K_v_* denotes the size of the convolution kernel; *A_k_* represents the adjacency matrix, *η* is a normalized diagonal matrix; where *M* is a learnable weight matrix and denotes element-wise multiplication.

In the temporal dimension, the implementation of graph convolution operations exhibits significant similarities to classical 2D convolution. Each vertex, corresponding to nodes in adjacent frames within a temporal sequence, maintains a fixed topological structure—specifically, each vertex possesses two adjacent nodes on the temporal axis. Consequently, after performing spatial graph convolution, applying 2D convolution to the temporal output feature maps effectively extracts temporal features, thereby achieving spatiotemporal graph convolution. ST-GCN introduces a weighted adjacency matrix generated through element-wise multiplication of the original adjacency matrix and a learnable weight matrix. The model employs spatial graph convolution and temporal convolution it first aggregates spatial features of skeletal joints per frame using the weighted adjacency matrix, then performs temporal convolution on these spatial features to extract temporal dynamics, enabling comprehensive spatiotemporal feature modeling of skeleton sequences. Compared to traditional CNN, this approach effectively handles non-Euclidean domain data, capturing skeletal dynamics and spatial characteristics.

### 3.4 Fusion of spatio-temporal and self-attentive graph convolutional networks BCST-GCN

To characterize the behavioral dynamics of individual pigs, the spatiotemporal feature sequences are subsequently input into the improvedST-GCN. As shown in Table 3, all experiments set the batch size to 64. The Stochastic Gradient Descent (SGD) algorithm is employed for optimization, with cross entropy loss used for backpropagation. The learning momentum, learning rate, and weight decay are configured as 0.9, 0.1, and 1e-4, respectively, with a spatiotemporal kernel size of 3 × 9. Each ST-GCN unit incorporates a feature residual fusion mechanism to enable cross-regional feature integration, while a dropout probability of 0.5 is applied to each unit to mitigate the risk of model overfitting. Finally, the generated feature vectors are fed into a SoftMax classifier to output motion classifications.

**Table 3:** BCST-GCN parameter settings.

Parametric	Value
Batch size	64
Epochs	100
Initial learning rate	0.1
Momentum	0.9
weight decay	0.0005

However, the fixed topology of ST-GCN limits the scope of inter-joint message transmission. This study analyses the limitations of ST-GCN in pig behavior recognition and incorporates a graph convolution method that combines global and local self-attention to enhance its adaptability to behavior dependent and dynamic features. The fixed topology structure exhibits two key shortcomings in modeling body part information transmission: First, it cannot capture non-physical associations. ST-GCN constructs skeletal graphs based on physical structures, describing direct node connections but struggling to represent latent cross-node interactions. For example, the synergistic interaction between the head and forelimbs during the “ground rooting foraging” behavior is not reflected in the graph topology. Second, it overly relies on fixed graph structures. In ST-GCN, the node features are learned through interactions with neighboring nodes, and the setting of the weights is as shown in Equation (2):

$${\bar{A}}_{k}=\eta^{-\frac{1}{2}}A_{k}\eta^{-\frac{1}{2}}$$ (2)

Here, *A_k_* is the adjacency matrix, *η* is the normalized diagonal matrix.

This mechanism overlooks the dynamic variations in inter-node interaction intensities, limiting its capacity to represent complex behaviors. Consequently, the message transmission mechanism heavily relies on graph structure while neglecting connection strengths between joints and their first-order neighbor joints. Behavior recognition depends on dynamic interactions between nodes and their neighbors; however, fixed topologies struggle to adapt to the diversity of pig behavioral characteristics.

The self-attention mechanism calculates neighborhood weights between any two nodes in the skeletal graph during node feature learning. The algorithm for the self-attention mechanism is described as follows:

Let the set of input node feature vectors be denoted as $h=\{h_{1},,h_{2},,..\ldots h_{n}\},h_{k}\in R^{F}$ , where n represents the number of nodes and *F* represents the dimension of node features. By performing a linear transformation operation on the feature vectors of h , $W_{h}$ is generated, with the node dimension being elevated from *F* to *F*′. This operation aims to expand the low-dimensional features into a high-dimensional space, thereby enhancing the feature representation capability.

In the above Formula (3), *e_kl_* represents the self-attention coefficient. *h_k_* and h_l_ are the feature representations of nodes *k* and *l* respectively, and ‖ indicates the concatenation operation of the two features. After *Wh_l_* and *Wh_k_* are concatenated, they are multiplied by the transpose of the weight matrix *A*. Finally, the LeakyReLU activation function is applied. It is normalized into a probability distribution via the SoftMax function to obtain the self-attention weight *a_kl_*, as shown in Equation (4).


$$e_{\mathrm{kl}}=\text{LeakyRelu}(a^{T}[Wh_{k}∥Wh_{l}])$$ (3)

$$a_{\mathrm{kl}}=\text{softmax}(e_{\mathrm{kl}})$$ (4)

To address the limitation of fixed topological structures in swine skeletal frameworks, a self-attention mechanism is introduced into the graph convolutional layer. The global self-attention matrix dynamically captures potential dependencies between joints and adjusts the graph topology, while the local self-attention matrix quantifies the physical connection strength between joints and their first-order neighbors. Both matrices synchronously update their parameters with the spatio-temporal convolution module and are continuously optimized during training. Following the integration of the self-attention mechanism, the graph convolution formula is updated from Equations (1)–(5).


$$a_{\mathrm{kl}}=\text{softmax}(e_{\mathrm{kl}})$$ (5)

Where, *W_k_* represents the weight matrix, *B_k_* is the local self-attention matrix, and *C_k_* represents the global self-attention matrix. *A_k_* is the adjacency matrix and *η* is the normalized diagonal matrix.

For the fixed topology in the problem, the global self-attention matrix is calculated in each network layer of ST-GCN according to the above steps, which is independent of the graph structure by learning from different action samples, and focuses more on the dependencies of non-physically connected edges. As shown in Equation (5), the skeleton graph achieves topological update by introducing *c* module, retaining physical edges while adding non-physical connected edges with strong dependencies. In order to solve the over-dependence of GCN node message passing on graph structure, a self-attention mechanism is introduced to calculate the connection strength between nodes and first-order neighbors. The local self-attention matrix *B_k_* is obtained, where *B_ij_* represents the self-attention weight between nodes *i* and *j*. As shown in Equation (5), the dot product of *A_k_* and *B_k_* reflects that the message passing of nodes is not only dependent on the graph structure, but also affected by the strength of physical connected edges. To simplify the newly generated graph topology, a threshold value *C_λ_* is introduced, as shown in Equation (6).


$$C_{\mathrm{ij}}=\{\begin{array}{c} C_{\mathrm{ij}},C_{\mathrm{ij}}>C_{\lambda }\\ 0,C_{\mathrm{ij}}<C_{\lambda } \end{array}$$ (6)

To optimize the computational efficiency and representational capacity of the ST-GCN network architecture, this study proposes a structural streamlining strategy to address hierarchical redundancy. The original model employs a multi-layer composite design in the spatial dimension, where layers 2–4, 6–7, and 8–10 form three consecutive modules with constant feature dimensions, resulting in parameter redundancy and computational bottlenecks. Through topological structure analysis, complete isomorphism was identified between layers 7, 9, and their preceding layers. By removing these redundant hierarchical structures, the model successfully achieves coordinated optimization of lightweight design and computational efficiency while maintaining spatio-temporal feature extraction capabilities.

## 4 Evaluation metrics

The Root Mean Square Error (RMSE) is a widely used metric for evaluating the prediction accuracy of regression models, primarily employed to quantify the discrepancy between predicted values and actual observations. Mathematically, RMSE is calculated by squaring the prediction error for each sample, computing the mean of these squared errors, and finally taking the square root of this average. Its mathematical expression is shown in Equation (7). To quantitatively evaluate the prediction performance of the proposed model, this section adopts the Root Mean Square Error (RMSE) for model evaluation and explains its definition and calculation. As a classic metric for assessing the prediction accuracy of regression models, RMSE is used to quantify the deviation between predicted values and actual observations, and its mathematical expression is given as follows. Here, n represents the number of samples, $y_{i}$ is the actual value of the i sample, and ${\hat{y}}_{i}$ is the predicted value of the i sample. A smaller RMSE value indicates a smaller discrepancy between the model’s predictions and the actual data, signifying higher predictive accuracy. Conversely, a larger RMSE value suggests a greater prediction error of the model. In the subsequent experiments, the RMSE metric is adopted for quantitative analysis and comparison to verify the effectiveness of the proposed optimization method.


$$\text{RMSE}=\sqrt{\frac{1}{n}\sum_{i=1}^{n}{\left(y_{i}-{\hat{y}}_{i}\right)}^{2}}$$ (7)

In this study, we employed accuracy, precision, recall, and F1-score as evaluation metrics to assess model performance. Accuracy serves as a fundamental indicator for evaluating classification model performance. It is calculated based on the matching degree between model predictions and ground truth labels. A prediction is considered correct when it matches the true label; otherwise, it is deemed incorrect. The formula is shown as Equation (8):


$$\text{Accuracy}=\frac{\mathrm{TP}+\mathrm{TN}}{\mathrm{TP}+\mathrm{TN}+\mathrm{FP}+\mathrm{FN}}$$ (8)

Here, True Positive (TP) denotes correctly predicted positive instances, True Negative (TN) represents correctly predicted negative instances, False Positive (FP) indicates negative instances incorrectly predicted as positive, and False Negative (FN) refers to positive instances incorrectly predicted as negative. Precision measures the proportion of correctly predicted positive instances among all samples predicted as positive, as detailed in Equation (9).


$$\text{Accuracy}=\frac{\mathrm{TP}+\mathrm{TN}}{\mathrm{TP}+\mathrm{TN}+\mathrm{FP}+\mathrm{FN}}$$ (9)

Recall measures the proportion of true positive cases correctly identified by the model. It reflects the model’s capability to detect all positive instances, where higher values indicate better performance in recognizing positive cases. The calculation formula is shown in Equation (10).


$$\text{Recall}=\frac{\mathrm{TP}}{\mathrm{TP}+\mathrm{FN}}$$ (10)

The F1-score represents the harmonic mean of precision and recall, serving as a balanced metric that prevents overemphasis on either precision or recall while providing a comprehensive evaluation of model performance. The calculation formula is shown in Equation (11).


$$F1\text{score}=\frac{2\text{PrecisionRecall}}{\text{Precision}+\text{Recall}}\times 100\%$$ (11)

## 5 Experimental results

### 5.1 Posture estimation test results

**Figure 7:**
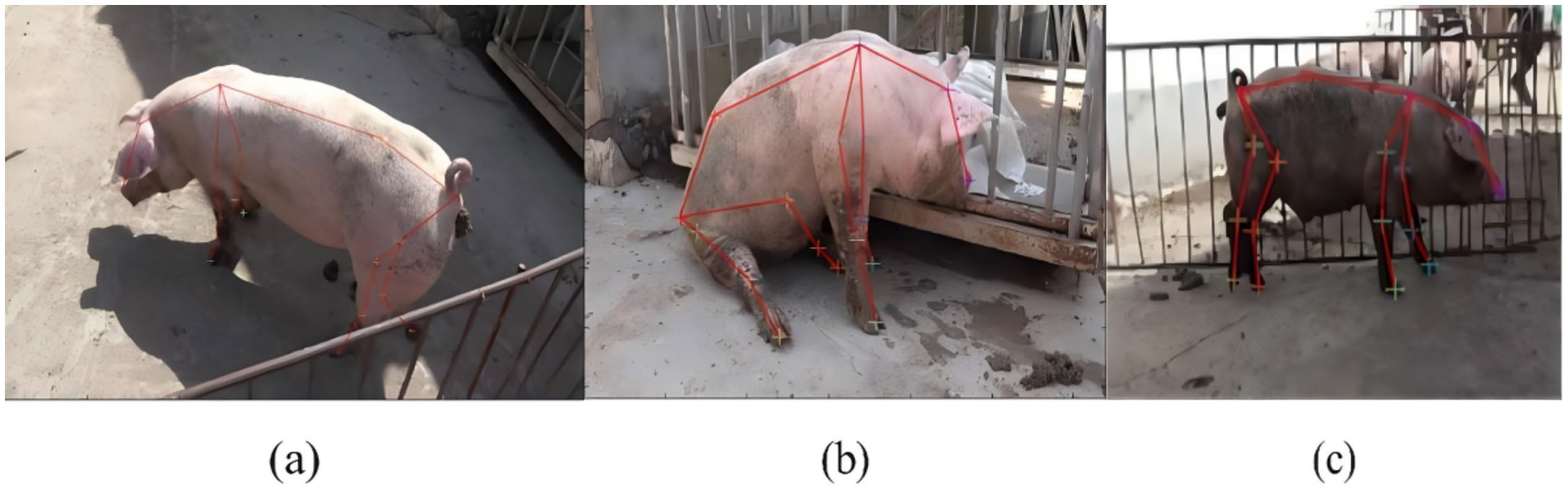
Skeleton diagrams of three typical behaviors: **(a)** foraging, **(b)** sitting, and **(c)** walking, respectively.

The specific experimental parameters are detailed in Table 4. In this study, the image dimensions were set to 1920 × 1,080 pixels, with a threshold set to 0.6 to balance prediction accuracy and false positive rates. During training, status information was logged and model weights were saved every 1,000 iterations, with the maximum iteration count set to 10,000 as the training termination criterion. Figure 7 demonstrates the skeletal characteristics of experimental subjects under three behavioral patterns: (a) During locomotion, the skeletal structure captures limb movement trajectories and displacement features, reflecting coordinated motion between the torso and extremities. (b) The Canine-like sitting behavior of pigs posture exhibits characteristic positioning with hindlimb flexion, torso reclination, and posterior weight shift. (c) The standing/walking posture displays symmetrical limb distribution while maintaining stable spatial relationships between the head and torso.

**Table 4:** DeepLabCut experimental parameters.

Parameter name	Parameter value
Image size	1,920 × 1,080
Thresholds	0.6
Show iterations	1,000
Saved iterations	1,000
Maximum iteration	10,000

**Figure 8:**
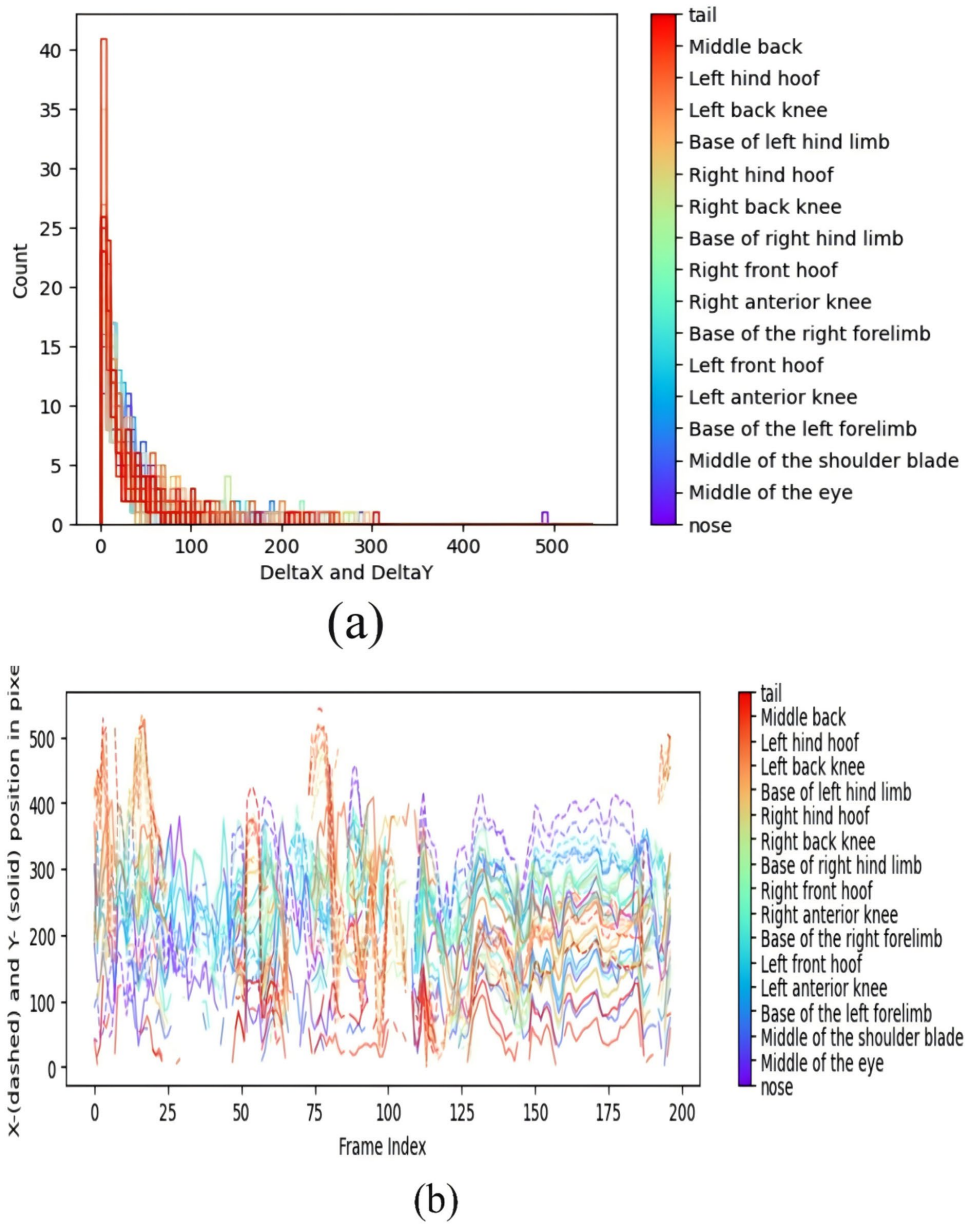
Results of canine—like sitting behavior of pigs behavior. **(a)** Behavioral histogram. **(b)** Walking trajectories.

Pig behaviors consist of continuous time-domain action signals with variable durations and spatiotemporal causal relationships. Relying solely on positional information would result in the loss of temporal characteristics. Therefore, analyzing the spatiotemporal trajectories of pig body parts provides critical behavioral insights. Taking the Canine-like sitting behavior of pigs posture as an example, Figure 8 presents representative results generated by DLC, demonstrating key kinematic features of this behavior. Figure 8 Results of Canine-like sitting behavior of pigs behavior (a) Behavioral histogram (b) Walking trajectories.

Figure 8a presents a histogram of pig Canine-like sitting behavior of pigs behavior, where the axes “DeltaX and DeltaY” represent the positional variation differences (2D coordinate changes) of body parts, and “Count” denotes frequency (with distinct colors for different body parts). Most body parts show positional variations concentrated within 0–100 units, with decreasing frequency as variation magnitude increases, exhibiting right-skewed distribution -indicating minimal positional adjustments during sitting. Figure 8b displays trajectory plots of walking behavior, with “Frame Index” as the temporal sequence and the vertical axis showing body part positions along X/Y coordinates. As frame index increases:

After completing 10,000 iterations, the pose estimation network model was evaluated on both the training and test sets, yielding the final keypoint detection accuracy and mean squared error (MSE) for each keypoint as shown in Table 5. Detailed analysis of the data revealed that the tailbase exhibited the smallest detection error among all keypoints, while the scapular midpoint and hooves (both fore and hind, left and right) also demonstrated relatively low errors. This error distribution pattern is physiologically reasonable since the tailbase is rarely occluded during pig movement, allowing stable feature capture during data collection and model analysis, thereby reducing errors. The hooves, being low-positioned and protruding structures, serve weight-bearing and locomotor functions with distinctive motion patterns and stable morphology, making them less susceptible to occlusion and enabling more accurate detection and localization by the model, resulting in smaller errors. This error distribution provides crucial reference data for studying the accuracy and optimization directions of pig pose estimation models. Furthermore, we calculated the root mean square error (RMSE) values for different body regions to further assess model performance.

**Table 5:** Key point localization errors in pigs.

Key point name	(RMSE)
1. Nose tip	6.72
2. Midpoint between ears	7.13
3. Cervical region	5.17
4. Midline between scapulae	4.98
5. Left forelimb proximal base	5.26
6. Left forelimb carpus	3.87
7. Left forehoof	4.01
8. Right forelimb proximal base	5.22
9. Right forelimb carpus	4.16
10. Right forehoof	4.58
11. Right hindlimb proximal base	5.23
12. Right stifle	5.43
13. Right hindhoof	4.69
14. Left hindlimb proximal base	5.14
15. Left stifle	5.05
16. Left hindhoof	4.58
17. Tail base	3.36

Among various backbone networks provided by DLC, this study compared the performance of ResNet-50, ResNet-101, MobileNet-v2-1.0, EfficientNet-b0, and EfficientNet-b3. The experimental results show that although MobileNet-v2-1.0, EfficientNet-b0, and EfficientNet-b3 are lighter in weight, they exhibit higher training and testing errors in the keypoint detection task, with overall accuracy lower than that of the ResNet series. The EfficientNet series models are slightly more accurate than MobileNet-v2-1.0, but still inferior to ResNet-50. Considering keypoint detection accuracy, model complexity, and inference efficiency, ResNet-50 is finally selected as the optimal backbone network. As shown in Table 6, ResNet-50 achieves a training error of 6.57 pixels and a testing error of 5.26 pixels, which are the lowest among all compared models, making it more suitable for pig pose estimation.

**Table 6:** Comparison of training error and test error for different models.

Network model	Training error (pixel)	Testing error (pixel)
ResNet-50	6.57	5.26
RseNet-101	6.75	6.92
Mobile-v2-1.0	7.95	6.64
EfficientNet-b0	8.23	7.11
EfficientNet-b3	8.12	7.86

During model training, image preprocessing consisted of randomly scaling the shorter edge to dimensions ranging from 256 to 480 pixels followed by center cropping to a standardized 224 × 224 resolution. The learning rate strategy employed dynamic adjustment, beginning with an initial rate of 0.0005 which increased to 0.02 after completing 10,000 iterations to maintain optimal performance throughout various training stages. For keypoint detection quality control, we implemented an Object Keypoint Similarity threshold set at 0.6, systematically filtering out any keypoint predictions scoring below this established reliability threshold.

### 5.2 Ablation study

A comparative analysis was conducted on the improved methodology, beginning with experimental validation of the DeepLabCut + ST-GCN feasibility which demonstrated satisfactory performance. Building upon this baseline, the BC module integrating both global and local self-attention mechanisms was incorporated for enhancement, along with optimization of the ST-GCN layer configuration. All experiments were performed under identical conditions, with the comparative results quantitatively presented in Table 7.

**Table 7:** Ablation study results.

Model	Accuracy	Precision	Recall
DLC + ST-GCN	88.42	89.29	88.53
DLC + BC + ST-GCN	91.07	91.04	89.08
DLC + BC + ST-GCN + Delete layers	95.36	94.9	95.41

**Figure 9:**
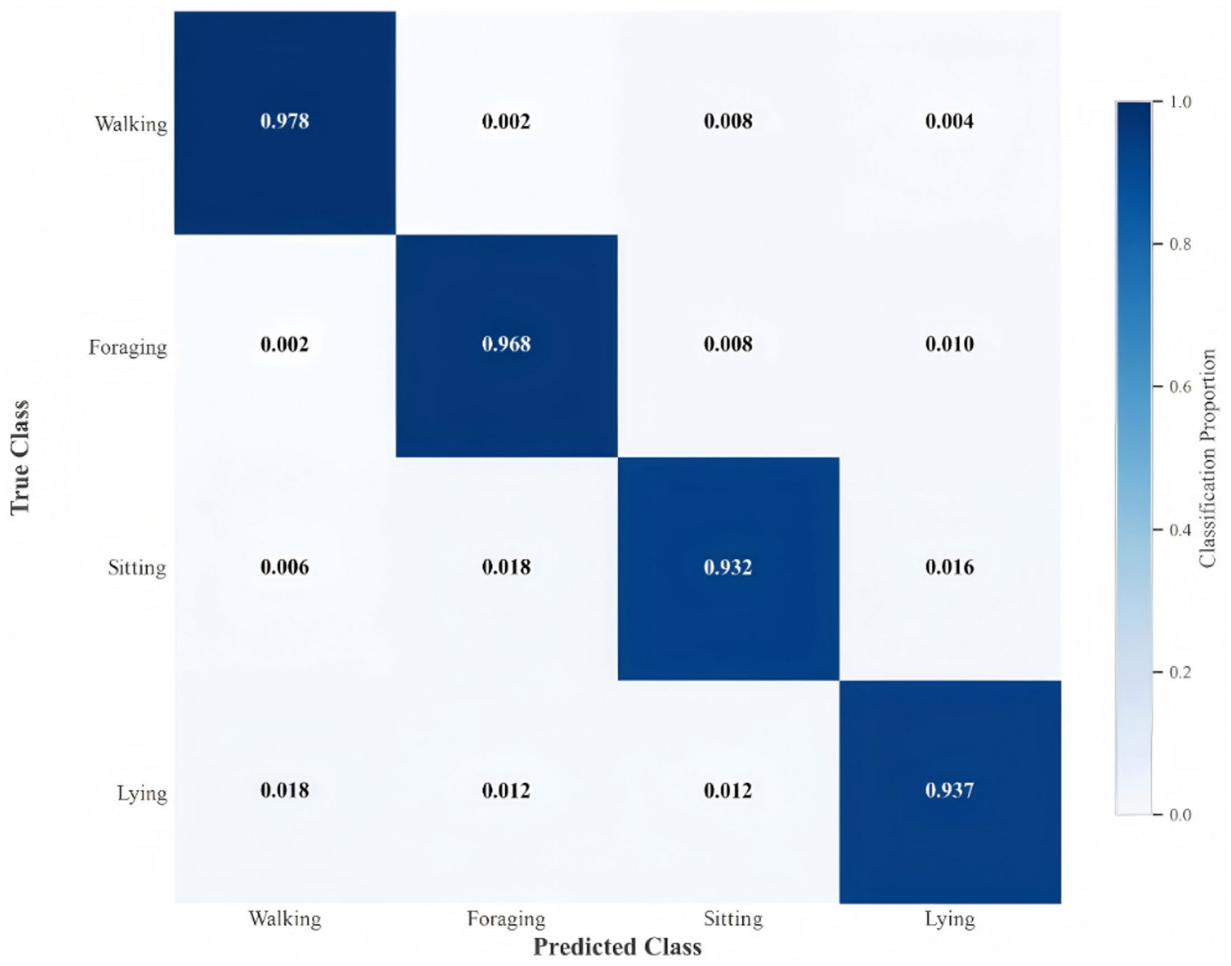
The confusion matrix of the model in this paper.

This paper analyzes the model classification results and inter-class confusion through the normalized confusion matrix shown in Figure 9. All values in the figure are presented as proportions ranging from 0 to 1.0, where the diagonal values represent the proportion of samples correctly classified into the corresponding category (i.e., the recognition accuracy of that class), and the off-diagonal values indicate the proportion of samples misclassified into the category corresponding to the column. The results show that the correct classification proportion for the Walking behavior is 0.978, with only 0.002, 0.008, and 0.004 of samples misclassified as Foraging, Canine Sitting, and Lying, respectively. The correct classification proportions for Foraging, Canine Sitting, and Lying are 0.968, 0.932, and 0.937, respectively. The model achieves high recognition accuracy for Walking and Foraging behaviors, but some confusion exists between Canine Sitting and Lying (e.g., 0.016 of Canine Sitting samples are misclassified as Lying, and 0.012 of Lying samples are misclassified as Canine Sitting), which may stem from the continuity of behaviors in real scenarios and feature overlap in transitional states.

To improve classification performance, this paper introduces a self-attention mechanism and an ST-GCN layer number optimization strategy. The improved model significantly increases the proportion of diagonal elements in the confusion matrix and reduces the values of off-diagonal elements, thereby effectively reducing inter-class confusion. Further combining layer number optimization, the final model achieves optimal performance, improving accuracy while reducing computational complexity. Experimental data show that the accuracy, precision, and recall of the final optimized model are increased to 95.61, 91.33, and 91.14%, respectively, verifying the effectiveness of the optimization strategy.

To reduce computational overhead while maintaining or even enhancing recognition performance, we performed a targeted optimization of the BCST-GCN network architecture. During experiments, quantitative analysis of the feature activation levels, gradient contributions, and feature effectiveness across network layers identified layers 3 and 5 as redundant. The feature maps output by these layers exhibited high redundancy with those of adjacent layers, contributed minimally to behavioral feature extraction, and their removal would not disrupt the overall feature propagation path or the network’s spatiotemporal feature fusion and dynamic modeling capabilities. Consequently, these redundant layers were pruned, leading to a significant reduction in computational operations.

The optimized architecture achieved a comprehensive performance improvement: the number of parameters decreased from 2.08 million to 1.98 million; using the original ST-GCN as the baseline, the accuracy increased from 92.31 to 95.07% (a gain of 2.76 percentage points); the inference time was reduced from 18.6 ms/frame to 15.2 ms/frame, representing an 18.3% improvement in efficiency; and the floating-point operations (FLOPs) were significantly reduced from 40.5 GFLOPs to 30.3 GFLOPs. Removing the redundant layers effectively lowered the computational burden, accelerated inference, and mitigated interference from feature redundancy, allowing the model to focus more on extracting critical features of pig behaviors. The results, summarized in Table 8, demonstrate a notable enhancement in the model’s overall performance.

**Table 8:** Parameter count comparison.

Model	Parameters/M	Accuracy/%	Inference time (ms/frame)	Computational cost (FLOPs)	Source
ST-GCN (29)	2.08 M	92.31	18.6	40.5	AAAI 2018
BCST-GCN	1.98 M	95.07	15.2	30.3	This work

### 5.3 Comparative experiments

All experiments in this study were conducted under strictly controlled and consistent conditions to ensure the fairness and comparability of the comparative results. All models were trained and tested based on a unified hardware and software environment, including an AMD Ryzen 7 5800X Central Processing Unit (CPU), an NVIDIA GeForce RTX 3060 Graphics Processing Unit (GPU), 32 Gigabytes (GB) of Random Access Memory (RAM), as well as Python 3.8, PyTorch 1.12.1, and Compute Unified Device Architecture (CUDA) 11.3. The same dataset splitting strategy, preprocessing pipeline, and data augmentation methods were adopted for all models. The training hyperparameters of each comparative model strictly followed the optimal configurations reported in their original literatures: the Parallel Multi-branch Spatiotemporal Convolutional Network (PMB-SCN) model utilized the Stochastic Gradient Descent (SGD) optimizer with an initial learning rate of 0.01, a momentum of 0.9, a weight decay of 0.0001, a batch size of 32, and was trained for 100 epochs with a dropout rate of 0.5, using the cross-entropy loss function; for the AlphaPose + Spatiotemporal Graph Convolutional Network (ST-GCN) pipeline, the AlphaPose module was trained with the SGD optimizer (learning rate = 0.1, batch size = 32) for 500 epochs, while the ST-GCN module employed the Adaptive Moment Estimation (Adam) optimizer (learning rate = 0.001, batch size = 16) for 100 epochs with a dropout rate of 0.5; the CNN + Long Short-Term Memory (LSTM) model was trained with the Adam optimizer (learning rate = 0.001), a batch size of 64, for 80 epochs, a weight decay of 0.0005, and a dropout rate of 0.5, also using the cross-entropy loss function. Model performance was uniformly evaluated and compared using accuracy, precision, recall, and the F1-score.

To objectively verify the advantages of the proposed model, this study selected existing pig behavior recognition methods, such as AlphaPose+ST-GCN (55), PMB-SCN (56), CNN + LSTM (57), and Time-series Neural Network (TNN) (58), for comparative experiments, and validated their applicability on the dataset constructed in this study. Although the work of Zhang et al. (55) verified the feasibility of combining spatiotemporal information and deep learning for livestock and poultry behavior recognition, their experiments were limited to a single site, a small population, and a night semi-farming scenario. In addition, their skeleton keypoint detection suffered from insufficient accuracy in some body parts, and core behavioral features were abandoned after improvement, making it difficult to form stable and effective feature representations. The model in Li et al. (56) was trained and tested only on high-end laboratory hardware, without quantifying key engineering indicators such as inference speed and floating-point operations, and lacked verification of deployment adaptability on commonly used equipment in farms. The core limitation of Chen et al. (57) is that neither skeleton extraction nor pose estimation was adopted, and modeling relied only on pixel-level temporal features of video frames, which failed to capture the structural skeleton information underlying pig behaviors. The main shortcoming of Zhang et al. (58) is the over-reliance on global feature extraction without targeted capture of local key features. When pig behaviors are close to the background, located at image edges, or involve complex local details, global features are easily disturbed by redundant information, making it hard to accurately locate core behavioral features and resulting in degraded recognition performance.

In this paper, a pig behavior recognition method combining pose estimation and the Spatial–Temporal Graph Convolutional Network (ST-GCN) is proposed. We compare our method with approaches that only introduce temporal information in deep learning, methods that do not utilize skeleton information, and methods that fail to fully explore the relationship between local joints and global features, so as to verify the effectiveness of the proposed method. Table 9 presents the comparative results of each model in terms of accuracy, precision, recall, and F1-score.

**Table 9:** Comparative experiments.

Network	Accuracy rate	Precision rate	Recall rate	F1
AlphaPose + ST-GCN (55)	89.20	83.30	85.60	84.40
PMB-SCN (56)	85.83	82.26	82.75	82.42
CNN + LSTM (57)	88.62	89.68	87.08	88.36
TNN (58)	91.93	88.36	89.92	89.13
Ours	95.36	94.9	95.4	95.1

**Figure 10:**
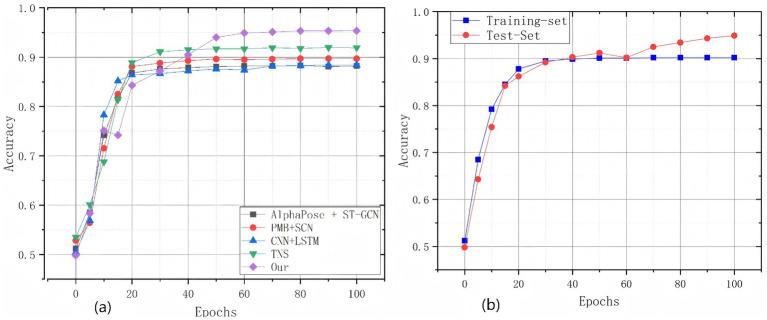
**(a)** Accuracy of the comparison model. **(b)** Accuracy of the training and test sets of the model in this paper.

**Figure 11:**
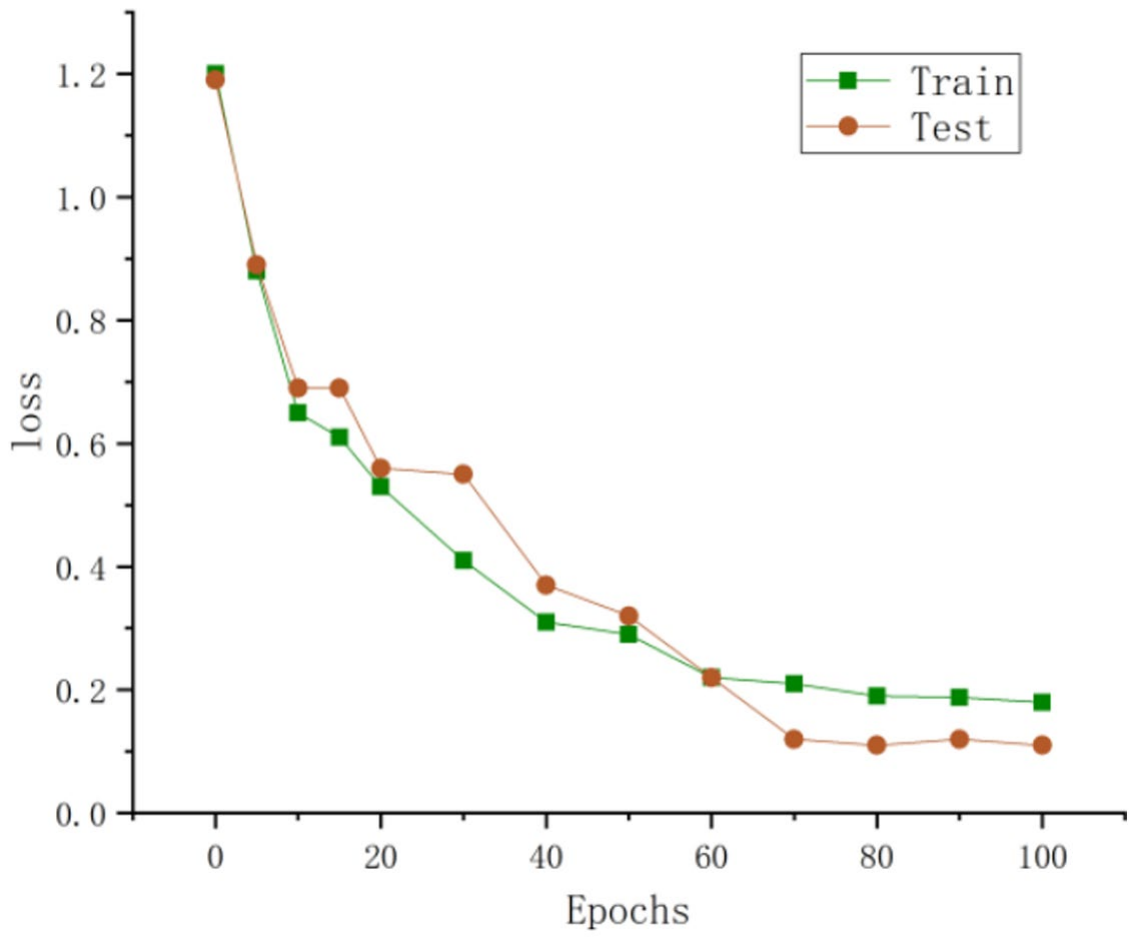
Loss values during training and testing.

The tabular data present a performance comparison of different models in behavior recognition tasks. The experimental results indicate that the PMB-SCN method achieves an accuracy improvement of 1.59% compared with AlphaPose + ST-GCN (55), but its overall performance is still lower than that of several subsequent methods. The CNN + LSTM (57) method outperforms AlphaPose + ST-GCN (55), and exhibits significant advantages with a precision of 89.68% and a recall of 87.08%. In contrast, the proposed method achieves the highest performance across all metrics, demonstrating an average accuracy of 95.36%, precision of 94.90%, recall of 95.41%, and F1-score of 95.10%. These results confirm that the proposed method enhances positive-class sample capture and reduces misclassification through optimized model architecture while maintaining overall high performance. The test set accuracy further reaches 95.36%. Figures 10, 11 illustrate the training dynamics and performance evaluation of the spatiotemporal graph convolutional network on the test dataset.

Figure 10a shows the evolution of accuracy of multiple models such as “AlphaPose + ST-GCN”, “PMB-SCN”, “CNN-LSTM”, “TNN”, and “Our” during the training process. The horizontal coordinate is the training rounds, and the vertical coordinate is the Accuracy. Curves with different labels and colors represent each model, respectively. At the beginning of training, the accuracy of each model is significantly improved with the increase of training rounds, which is a powerful representation that the model can efficiently extract features from the training data and is in the stage of rapid convergence. With the continuous progress of training, the growth rate of accuracy gradually slows down, which means that the model transitions to a relatively stable learning state and the accuracy rate becomes stable. Among them, “Our” model always maintains a high accuracy level during the whole training cycle, and has obvious advantages over other competitive models. This phenomenon shows that the model has a good performance when dealing with the current task. Figure 10b illustrates the accuracy dynamics of our model on the training and test sets across epochs. Both curves rise rapidly in early training and stabilize afterward, with the test-set accuracy slightly exceeding the training-set accuracy in later stages, demonstrating the model’s strong generalization ability.

**Figure 12:**
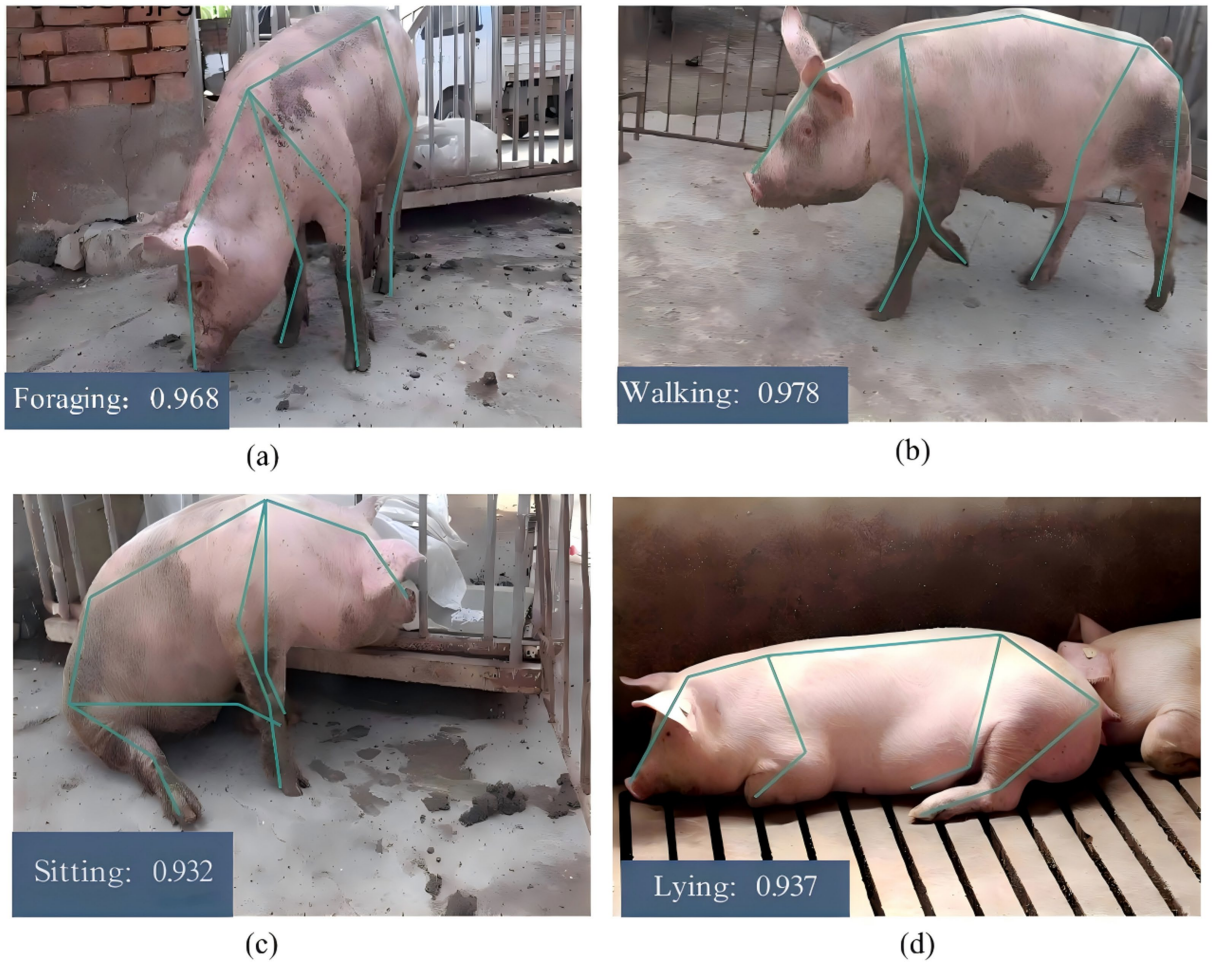
Behavior recognition results. **(a)** Foraging, **(b)** walking, **(c)** sitting, **(d)** lying.

As shown in Figure 11, the training loss curve exhibits a pronounced downward trend and converges clearly, whereas the test loss curve remains stable with minor fluctuations. This consistent trend validates the efficient training process and strong generalization capability of the proposed model. Furthermore, as illustrated in Figure 12, the model achieves superior performance in pig behavior recognition, and the results verify the effectiveness and feasibility of the improved spatiotemporal graph convolutional network for identifying fundamental behaviors in farmed pigs.

## 6 Conclusion

This study introduces an enhanced ST-GCN model tailored for pig behavior recognition. The improved architecture integrates a self-attention mechanism into spatial convolutions while reducing network depth, thereby achieving higher accuracy compared to the original framework.

To validate the performance of the BCST-GCN model, this study conducted ablation experiments. The experimental design included five different models for comparative analysis: a pose estimation module for skeleton extraction serving as the prerequisite for pig behavior recognition, and four improved variants of the ST-GCN behavior recognition network with incorporated self-attention mechanisms and optimized layer configurations while maintaining the same pose estimation module. Experimental results demonstrate that after targeted improvements, the proposed method outperformed the other four models in terms of precision, accuracy, and recall.

Under identical experimental conditions, this study compared the improved model with AlphaPose + ST-GCN, PMB-SCN, CNN + LSTM, and TNN models. On our dataset, BCST-GCN demonstrated superior performance. The experimental results confirm that the proposed BCST-GCN model achieves higher recognition accuracy, providing a reliable and efficient solution for fundamental pig behavior recognition.

## 7 Discussion

To meet the demand for behavior recognition in large-scale pig farming, this study proposes an improved ST-GCN model (BCST-GCN) dedicated to pig behavior recognition. Based on the original ST-GCN, the model integrates a self-attention mechanism into the spatial convolution module to strengthen the ability to capture key skeletal features of pigs. Meanwhile, redundant network layers are selectively reduced to optimize the model structure, improve recognition accuracy, and balance the requirements of feature extraction and engineering deployment.

To verify the model performance, multiple groups of experiments are designed in this study. Pig skeleton extraction using the pose estimation module is conducted as a pre-processing step. Ablation experiments are performed to compare the performance of different improved schemes. The results confirm that the BCST-GCN combining self-attention mechanism and layer optimization achieves the best performance, and is significantly superior to the control groups in core indicators including precision, accuracy, and recall, verifying the effectiveness and pertinence of the proposed improvements.

Compared with state-of-the-art pig behavior recognition models such as AlphaPose+ST-GCN and PMB-SCN, BCST-GCN exhibits superior performance on the dataset used in this study, demonstrating that it can provide an efficient and reliable technical solution for intelligent pig behavior recognition in large-scale farming.

Although the BCST-GCN model achieves satisfactory recognition accuracy, it still has limitations for practical application. The model is currently only tested on data of single pigs; problems such as body occlusion and individual interaction in group-housed environments have not been solved, and the recognition performance in such scenarios needs to be verified.

In addition, this study conducts a quantitative analysis of the model’s engineering indicators: the baseline floating-point operations (FLOPs) are 40.5 GFLOPs, which are reduced to 30 GFLOPs after layer optimization, with the single-frame inference time shortened from 18.6 ms to 15.2 ms. The model does not demand high-end computing hardware and can reuse the existing monitoring and computing facilities in pig farms, significantly lowering deployment and data acquisition costs, thus rendering it suitable for large-scale application in small and medium-sized pig farms.

This study aims to provide technical support for the intensive and intelligent management of pig farming. The core value of the BCST-GCN model lies in the deep integration of skeleton-based behavior recognition technology with practical pig production and veterinary clinical practice. The model can accurately identify the rhythms and status of key behaviors such as lying, walking, feeding, and canine-like sitting in pigs, providing objective data support for breeding environment regulation, pig health evaluation, and the improvement of animal welfare. It can also effectively capture early behavioral changes including reduced feed intake, abnormal activity, and atypical postures, enabling non-contact intelligent early warning of sub-health, stress responses, and potential diseases in pigs. This helps farmers and veterinarians detect risks in a timely manner and optimize management decisions, thereby effectively improving the efficiency, health status, and welfare level of pig farming.

Future research will focus on practical application scenarios in commercial pig farms. Target detection and multi-object skeleton tracking algorithms will be integrated, and a skeleton occlusion completion strategy will be introduced to improve the recognition robustness of the model in complex group-housed environments. A lightweight edge-side deployment program will be developed to realize rapid integration with the existing monitoring systems of pig farms, so as to promote the implementation and in-depth application of intelligent recognition technology in livestock and poultry breeding.

## Data Availability

The raw data supporting the conclusions of this article will be made available by the authors, without undue reservation.
